# Acute haemothorax secondary to ibrutinib: A case report

**DOI:** 10.1002/jha2.367

**Published:** 2022-09-06

**Authors:** Jessica Sue Yi Wong, Shalal Sadullah

**Affiliations:** ^1^ Department of Haematology James Paget University Hospitals NHS Foundation Trust Great Yarmouth UK

Mantle cell lymphoma (MCL) is a subtype of non‐Hodgkin lymphoma that frequently follows an aggressive clinical course, with 5‐year overall survival (OS) ranging from 17% to 85% [1]. Previous management comprised intensive chemotherapy regimens with or without autologous stem cell transplantation, however this was limited by chemotherapy‐related toxicities especially in older patients. There has since been a trend towards the use of chemotherapy‐free treatments, with the aim of prolonging disease remission while minimising toxicity. Ibrutinib is an orally administered first‐generation Bruton's tyrosine kinase (BTK) inhibitor that was licenced as first‐line treatment during the COVID‐19 pandemic [2]. It binds to cysteine 481 within the ATP binding site of the BTK kinase domain. It also reacts with other TEC kinases (BMX, ITK, BLK) distributed in various tissues, resulting in side effects such as atrial fibrillation and bleeding [3, 4, 5]. Here, we report a case of acute haemothorax in a patient with MCL, 3 months after the initiation of ibrutinib as first‐line therapy.

## 1 CASE

A 79‐year‐old male with chronic obstructive pulmonary disease, hypertension and hepatitis C was diagnosed with MCL incidentally after the finding of small lymphoid cells with round irregular nuclei, expressing CD20+, PAX5+, CD10–, BCL6–, BCL2+, CD5–/weak+, cyclin D1+, CD23–/occasionally weak+, CD21 weak+, CD43 weak+ and CD3– on a prostatic biopsy taken as part of the work‐up for suspected prostate cancer. Since he was asymptomatic and blood parameters (haemoglobin 139 g/L, white cell count 13.3 × 10^9^/L, platelets 304 × 10^9^/L, lymphocytes 7.32 × 10^9^/L) were within the normal range, a watch‐and‐wait approach was taken. One year after the diagnosis of MCL, the patient presented acutely to hospital with drenching night sweats and symptomatic anaemia. His admission blood tests revealed anaemia (haemoglobin 108 g/L) and lymphocytosis of 60.95 × 10^9^/L. Clotting studies were normal (INR 1.02 ratio, APTT 26.6 s, PT 11.8 s). Urgent CT chest abdomen and pelvis scan showed increasing neck, mediastinal and abdominal lymphadenopathy with new liver lesions indicative of significant disease progression. Following discussion in the multidisciplinary team (MDT) meeting and patient consent, he was commenced on ibrutinib 560 mg once daily. He developed an episode of bruising over his elbow after accidentally hitting it but did not have any other side effects from the ibrutinib. Three months later, he presented again with worsening dyspnoea and peripheral oedema, despite treatment for lower respiratory tract infection in the community. Chest radiograph revealed a large left‐sided pleural effusion; 700 ml pleural fluid was aspirated that was heavily blood‐stained. Cytological examination showed no malignant cells. The patient was not prescribed any antiplatelet agents or anticoagulants and denied any recent falls or chest trauma.

A repeat chest radiograph 1 week later demonstrated no significant change in the size of the effusion. A second pleural aspirate showed mainly B cells expressive oCD20, CD5 (weak), BCL2, and negative for CD3, CD10, BCL6, CD23, LEF1, SOX 11 and CD43. A small proportion of the cells showed a very weak staining with cyclin D1. B cells with weak cyclin D3 expression. Fluorescent in‐situ hybridisation (FISH) showed t(11;14)q(13;32), IDH‐CCNDI translocation in the tumour cells. Together these findings were in keeping with MCL. Gram‐stain and cultures tested negative. As he was clinically stable, he was discharged and advised to continue with ibrutinib. Serial outpatient chest radiographs showed a persistent left‐sided pleural effusion. A staging CT showed a large left‐sided pleural effusion with collapse and consolidation of the left lung, and reduction in lymphadenopathy below the diaphragm.

One month later, he was readmitted with severe dyspnoea. Chest radiograph showed increased volume of the left‐sided pleural effusion necessitating therapeutic thoracocentesis. Repeat CT scan showed increased pleural nodularity thickened walls surrounding the fluid collection. Subsequently, he underwent a thoracoscopy: two litres of haemorrhagic fluid were drained, which showed evidence of lymphomatous infiltration and pus cells consistent with superadded empyema. Following MDT discussion, ibrutinib was determined to be the causative agent and discontinued. He was not on any concomitant CYP3A4 inhibitors. Soon after stopping ibrutinib, the patient's breathing improved significantly. Consistent with this, staging CT 5 months later showed reduction in volume of the left pleural effusion, however there was new renal lymphomatous infiltration. He has since been commenced on rituximab‐bendamustine therapy for progressive MCL.

To our knowledge, this is the first reported case of acute haemothorax secondary to ibrutinib therapy for MCL. A case of spontaneous haemothorax in a patient on ibrutinib therapy for transfusion‐dependent chronic lymphocytic leukaemia (CLL) has been reported [6]. Minor bleeding events are known to occur commonly with the use of ibrutinib, however severe bleeding can be triggered by the concurrent use of anticoagulants and antiplatelets [7]. Caron et al. described a major bleeding incidence of 2.76 per 100 patient‐years, and overall bleeding incidence of 20.8 per 100 patient‐years in patients receiving ibrutinib [8]. Ibrutinib‐associated bleeding is thought to be related to multiple mechanisms – the downregulation of platelet transmembrane receptors, including the platelet collagen receptor glycoprotein VI, which results in a reduction in collagen‐mediated platelet aggregation; the interference with platelet GP1b‐mediated platelet functions and the inhibition of platelet adhesion to fibrinogen [9]. Warfarin and other vitamin K antagonists should not be administered alongside ibrutinib [10].

Patients should be counselled about the risk of bleeding prior to commencing treatment. Those on antiplatelets will need careful consideration of bleeding and cardiovascular risks. In cases of major bleeding, ibrutinib should be withheld and supportive management given using blood products. Platelet transfusions have been shown to reverse aberrant ibrutinib‐related haemostasis, although this needs to be assessed on a case‐by‐case basis in intracranial bleeding [5]. The decision whether to resume ibrutinib will depend on the index of suspicion for a drug‐related aetiology, and patient‐specific factors such as bleeding risk and the underlying disease. Clinicians should consider haemorrhagic pleural effusion as a differential for any patient presenting with a pleural effusion on ibrutinib.

## AUTHOR CONTRIBUTIONS

Jessica Sue Yi Wong wrote the original draft of the manuscript. Shalal Sadullah made the diagnosis and oversaw the care of the patient in the case. Both the authors read and approved the final manuscript.

## FUNDING INFORMATION

None.

## CONFLICT OF INTEREST

The authors declare no conflict of interest.
